# Chimpanzee Alarm Call Production Meets Key Criteria for Intentionality

**DOI:** 10.1371/journal.pone.0076674

**Published:** 2013-10-16

**Authors:** Anne Marijke Schel, Simon W. Townsend, Zarin Machanda, Klaus Zuberbühler, Katie E. Slocombe

**Affiliations:** 1 Department of Psychology, University of York, York, United Kingdom; 2 Budongo Conservation Field Station, Masindi, Uganda; 3 Animal Behaviour Group, Institute of Evolutionary Biology and Environmental Studies, University of Zurich, Zurich, Switzerland; 4 Department of Human Evolutionary Biology, Harvard University, Cambridge, Massachusetts, United States of America; 5 Department of Comparative Cognition, University of Neuchâtel, Neuchâtel, Switzerland; 6 School of Psychology & Neurosciences, University of St Andrews, St Andrews, United Kingdom; University of Sussex, United Kingdom

## Abstract

Determining the intentionality of primate communication is critical to understanding the evolution of human language. Although intentional signalling has been claimed for some great ape gestural signals, comparable evidence is currently lacking for their vocal signals. We presented wild chimpanzees with a python model and found that two of three alarm call types exhibited characteristics previously used to argue for intentionality in gestural communication. These alarm calls were: (i) socially directed and given to the arrival of friends, (ii) associated with visual monitoring of the audience and gaze alternations, and (iii) goal directed, as calling only stopped when recipients were safe from the predator. Our results demonstrate that certain vocalisations of our closest living relatives qualify as intentional signals, in a directly comparable way to many great ape gestures. We conclude that our results undermine a central argument of gestural theories of language evolution and instead support a multimodal origin of human language.

## Introduction

Understanding the evolutionary origins of language, one of humankind’s defining features, is a challenge that attracts considerable multidisciplinary research effort. One important line of evidence comes from comparative research on closely related species, which enables us to identify elements of language that are unique to the human lineage and elements that are shared with our primate relatives and that were therefore likely present in our common ancestors [1]. One critical and defining feature of language is that it is produced intentionally, however the evolutionary routes of this facet of language remain a matter of fierce debate.

Intentionality is a concept that is discussed across disciplines from philosophy to psychology but a cross-disciplinary consensus on a definition for intentionality and how to identify it remains elusive. One valuable scheme for considering the degree of intentionality underlying behaviour is provided by Dennett [2] and his different levels of intentionality can be usefully applied to the communicative behaviour of animals [3], [4]. Zero-order intentionality attributes no mentality to the individual and it is assumed their behaviour is a product of relatively automatic, reflexive processes (e.g. an animal produces alarm calls because it is frightened). First-order intentionality attributes beliefs and desires to the behaving individual, including a desire to modify or influence the behaviour of others (e.g. an animal produces alarm calls to get others to move away from a predator). At this level, although individuals may recognise the effect of their signals on another’s behaviour, they crucially need no understanding of the mind of the other individual. Second-order intentionality requires an individual to have an understanding of both their own and others’ mental states and for their behaviour to reflect a desire to modify another’s mental state, not just their behaviour (e.g. an animal produces alarm calls to inform an ignorant individual of a predator and thus change their knowledge state). Grice [5] argues that intentional communication requires both the signaller and receiver to take into account each other’s mental states and thus true communication requires a minimum of second order intentionality. Whilst human language requires second order intentionality or above, the level of intentionality inherent in the production of communicative signals in our primate cousins remains unclear.

Only a small number of studies have attempted to test whether primates produce their communicative signals to modify the mental states of others (second-order intentionality). In an early study macaque monkeys failed to modulate their calling behaviour in accordance with the ignorance or knowledge states of their offspring about either a danger or a food source [6], and this is in keeping with the generally poor performance of monkey species on theory of mind tests [4], [7]. In contrast, there is now evidence indicating that our closest living relatives can understand knowledge/ignorance states of others [8], [9], but the extent to which these skills influence signal production is still unclear. In one recent field study, wild chimpanzees produced alert hoos in response to a predator model at different rates depending on the presumed knowledge level of receivers [10]. Chimpanzees were presented with static snake models and the effect of both the caller’s and the receiver’s previous knowledge of the snake (ignorant, partial knowledge, full knowledge) on call production was examined. Although more calls were given when receivers were ignorant, in these cases the callers were also previously ignorant of the snake, so receiver knowledge was confounded with caller knowledge. The elevated call rate in these cases may therefore have simply reflected fear in the caller as they discovered the predator (zero-order intentionality). The critical analysis in this study was therefore one that showed that knowledgeable chimpanzees called more to receivers who had heard alert calls but not seen the snake (partially knowledgeable) compared to those who had seen the snake (knowledgeable). In this analysis, however, only a small subset of data was used where there was always a chimpanzee approaching the snake. An alternative explanation for these results is that calling was mediated by the behaviour of the chimpanzee approaching the snake, rather than their knowledge state. Individuals with less knowledge may have approached the snake with inappropriate speed or confidence and this incongruent behaviour may have been alarming in itself to the potential caller, triggering production of calls (zero order intentionality). Indeed, chimpanzees have been previously documented to produce fear responses to group members behaving in unusual or inappropriate ways (e.g. movement of individuals partially paralysed by polio [11]) and this alternative explanation for these results remains to be empirically tested. Thus, currently it seems premature to conclude that chimpanzee calling behaviour is mediated by an understanding of receiver mental states (second order intentionality), when lower level explanations have not been convincingly excluded.

Whilst there is currently no unequivocal evidence for second order intentionality in primate signal production, the question of whether primates even demonstrate first order intentionality during signal production is still a matter of debate [12]. Distinguishing between inflexible, automatic signal production elicited in a reflexive manner by specific stimuli (zero order intentionality) and voluntary signal production directed at a recipient to change their behaviour (first order intentionality) is important as first order intentionality is a necessary prerequisite for second order intentionality and ‘true’ communication [5]. Identifying even first order intentionality in non-linguistic beings is, however, a challenge.

Although there is a long tradition of examining goal-directed behaviour in a range of species, e.g. [13], [14], in the realm of communication it was developmental psychologists working with pre-linguistic infants who proposed an important set of behavioural criteria for distinguishing intentional from reflexive gestural signal production ([15], see Table 1). These criteria have since been successfully applied to great ape gestural communication (as reviewed in [16]). It is important to highlight that most of the criteria developed to identify intentional signalling in preverbal human infants and non-human primates (Table 1) do not necessarily indicate first order rather than zero order intentional signal production if applied in isolation: lower level and less cognitive explanations for these behaviours are available. For instance, audience effects could be mediated by differing arousal levels caused by the presence of others (e.g. social facilitation), and sensitivity to the attentional state of the recipient could represent a learned discrimination (e.g. only signal when recipient’s face is visible). Such lower-level explanations of associative learning or emotional processes are often applied post-hoc to findings to refute cognitive explanations of behaviour [17]. It is therefore important to look for convergent evidence from multiple criteria before evoking cognitive explanations of intentional signal production [18].

**Table 1:** Criteria used for identifying intentional production of communicative signals, as outlined in the study of great ape gestures [19]–[36].

Criteria	Explanation
Social use	The signal is directed at a recipient. This can be assessed at various levels:
	1. Presence/absence audience effect: the signal is only produced in the presence of a recipient.
	2. Composition of audience: the signal is only produced in the presence of certain recipients (e.g., kin, dominants, friends)
	3. Behaviour of audience: signal production is contingent on the behaviour of the recipient
Sensitivity to attentionalstate of recipient*	Visual signals are only produced in the field of view of recipients. If signaller does not have a recipient’s visual attention, tactile or auditory signals should be produced. This can also be considered a level (3) audience effect.
Manipulation of attentional state of recipient*	Before a visual signal is produced, attention-getting behaviours are directed towards a recipient who is not visually attending to the signaller.
Audience checking andgaze alternation	Signaller monitors the audience and visually orients towards the recipient before producing a signal. If a third entity is involved, gaze alternation may occur between recipients and this entity.
Persistence or elaboration	Goal-directed signalling shown by repetition of the same signal (persistence), or production of different signals (elaboration) until the desired goal is met.

* indicates applicable only to visual signals and therefore not relevant for vocal production.

Evidence from observations of conspecific interactions as well as experiments where great apes produce signals to human experimenters to request food have shown that great ape gestures can meet all of these behavioural criteria for intentionality [19]–[36]. As manual gestures are also considered, a priori, to be under voluntary control [37], great ape gestures are routinely characterised as intentional (e.g. [20]–[23], [32]–[34], [36]). The first order intentionality shown in great ape gesture production is argued to represent an important precursor to the second order intentionality required for language [12].

In contrast to great ape gestures, primate vocalisations have been traditionally characterized as unintentional and emotionally driven [12]. This apparent difference between the zero order and first order intentionality underlying primate vocalizations and great ape gestures has been used by some prominent researchers to support arguments that language originated from a gestural, rather than a vocal, system (e.g. [12], [38]–[43]). It is, however, possible that this apparent difference is due to the different approach, methods, and study species that primate gesture and vocal researchers have used [44]. In contrast to gestural research, intentionality has rarely been the focus of primate vocal research, thus it is vital that directly comparable evidence is gained to empirically test whether great ape vocal production engages first order intentionality.

Although no previous study has systematically applied the set of criteria used in gestural research to primate vocal production, there is some evidence showing that primate vocal signals can meet some of these criteria in isolation. A single study on Thomas langur monkeys demonstrated the capacity for goal-directed vocal production, as males persisted in alarm calling until all group members had vocally responded [45]. A more substantive body of evidence indicates that primate vocalizations are used socially, as demonstrated by sensitivity to the audience on a number of levels. Primates increase their vocal production in the presence of others [46], [47] especially kin [48] and important social partners [49], [50]. Captive chimpanzees also modulate vocal and gestural production as a function of the attentional state of a human they are begging from [29], [31]. In addition, there is growing evidence that primates have voluntary control over the initiation of vocalisations. On a behavioural level vocalisations can be suppressed (e.g. [51])and selectively produced in highly specific social circumstances (e.g. [52], [53]) and a range of primates have been successfully conditioned to produce vocalisations in response to arbitrary stimuli, reviewed in [54]. On a neurological level, cortical involvement in the production of vocalisations has also been shown in chimpanzees [55] and monkeys [56], which implies vocal production is influenced by cognitive processes.

**Figure 1:**
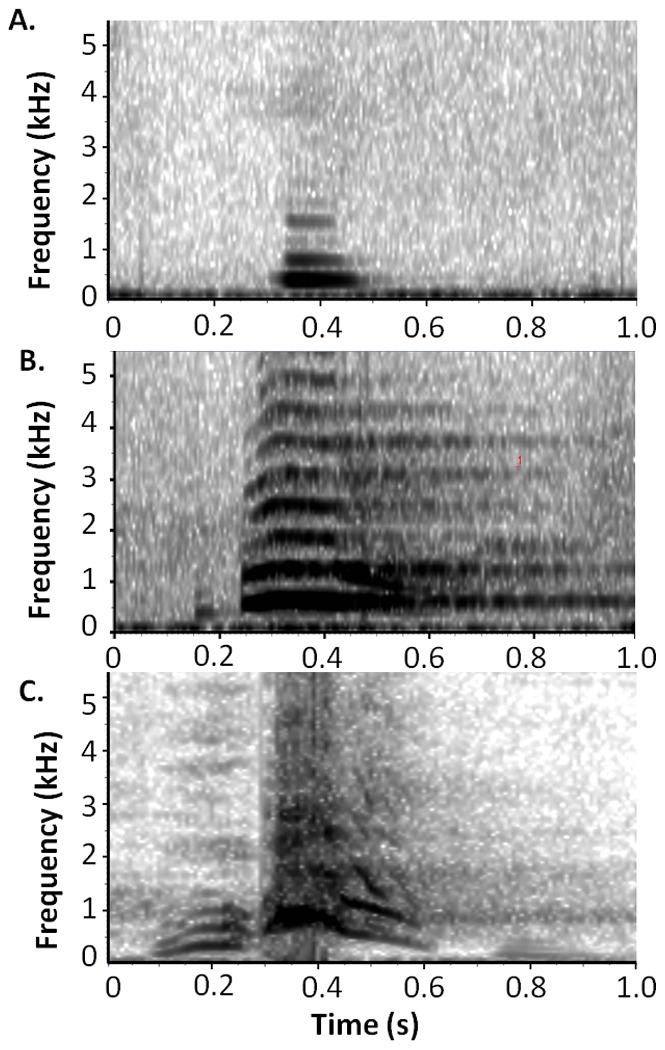
Example spectrographic representations of chimpanzee alarm calls. This Figure illustrates (A) Soft Huu (SH), (B) Alarm Huu (AH) and (C) Waa Bark (WB) vocalizations. The x-axis represents time in seconds, the y-axis frequency (KHz). The darkness of the image represents the amount of acoustic energy present, or the amplitude of the sound. SH are short (∼100 ms), tonal, and low pitched (<500 Hz), and usually have a low amplitude and little frequency modulation (Audio S1). These soft calls are unlikely to be heard by individuals further than 50 m from the call producer and are comparable with the ‘huu vocalizations’ described by Goodall [11] as well as the alert hoos reported in Crockford et al. [10]. AH are longer, louder, higher pitched, and with more frequency modulation compared to soft huus (Audio S2). These tonal calls are comparable with the ‘alarm calls’ described in Slocombe and Zuberbühler [72]. WB are loud, abrupt sounds with a noisy spectral quality (Audio S3). They typically start with a low frequency ‘w’ introduction at call onset, followed by considerable frequency modulation in the subsequent higher frequency element that can sound like an ‘aa’ ‘aow’ or ‘aoo’ sound to the human ear.

Despite previous research showing that initiation of vocalisations may be voluntary and single markers of intentionality may be present in primate vocal production, no previous study has tested the production of chimpanzee vocal signals across multiple markers of intentionality, in a comparable manner to chimpanzee gestures. In this study, we investigated whether wild chimpanzees of the Budongo Forest Reserve, Uganda, produced alarm calls in an intentional manner when encountering a predator model, by testing their vocal behaviour against multiple markers of first order intentionality (Table 1). Chimpanzees produce a variety of graded vocalizations in response to predators and in this study we distinguished ‘soft huus’ (SH), ‘alarm huus’ (AH) and ‘waa barks’ (WB; see Fig. 1; Audio S1, S2, S3).

In order to test if these calls were produced with first order rather than zero order intentionality, we presented focal individuals with a moving python model both when alone and as part of a social group and recorded their subsequent behaviour. We tested their vocal behaviour against three different markers of intentionality: (i) social use, (ii) audience checking and gaze alternation, and (iii) persistence (Table 1). If chimpanzees produced alarm calls intentionally as recipient-directed signals, we first expected to find that calls were used socially and we examined this on two different levels: most simply we examined if alarm calls were only produced in the presence of an audience. We then examined whether alarm calls were indiscriminately produced in the presence of others, or whether callers were sensitive to who was present and thus call production was mediated by the arrival of specific individuals. Secondly, if these calls were directed at others, we expected individuals to visually monitor recipients and to look at them before signalling. We also expected calls to be associated with gaze alternation between recipients and the snake. Finally, if signallers produced their calls with the goal of warning group members about the snake rather than simply expressing their own fear we expected to find that they persisted in calling until group members were safe (physically distant to the danger or aware of the ambush predator: Table 2).

**Table 2:** Behaviours continuously coded from when the snake was revealed to the end of the trial when the focal individual had moved further than 10

BEHAVIOUR CATEGORY	SPECIFICATION
Focal proximity to the snake	<1 m, 1–5 m, 5–10 m, >10 m
Position of the focal	Tree or Ground
Focal looking behaviour	Look at snake, look at other chimp, look at distant calls, look elsewhere. Looking direction was based on the head direction of the focal individual [21], which was observed from at least two different angles. Real-time commentary of their head direction was used in conjunction with the videos to determine their looking direction.
Focal Movement	Approach snake, move away from snake, move towards other chimp, no movement
Focal alarm calls	Soft Huu (SH), Alarm Huu (AH), Waa Bark (WB). Each call within a bout was coded as a point event.
Focal alarm calling bout	Sequence of calls of the same type: SH bout, AH bout, WB bout. A new bout was defined as a call given after 30 sec of silence or calls of a less ‘urgent’ type (SH<AH<WB) from the caller.
Non-focal calls	No calls others, within group calls, distant calls
Approach of non-focal individual to the snake	Non-focal individuals move towards the snake, no approach from non focal individuals
Risk assessment of non-focal individuals	All non focal individuals in party are safe: safety was defined as (1) awareness of the ambush predator (bipedal approach, visual searching of snake area, production of call) or (2) sufficient physical distance from the predator to not be in danger (up a tree or >10 m from the snake).At least one non-focal individual is in danger (not aware of ambush predator – no calls, bipedal approach or visual searching of snake area and physical proximity to the snake <10 m on the ground)

## Methods

### Ethics Statement

The Ethical review board of the Department of Psychology at the University of York approved the protocol for this experimental field study with wild chimpanzees. Permits to conduct our work with the chimpanzees at the Sonso field site of the Budongo Conservation Field Station in Budongo Forest Reserve were obtained from the Uganda National Council for Science and Technology (NS263), the Uganda Wildlife Authority (RES50), and the President’s Office.

### Study Site

This study was conducted with the habituated Sonso chimpanzee community [57] of the Budongo Forest Reserve, Uganda (1°35′ and 1°55′N and 31°08′ and 31°42′E), between January 2010 and December 2011. The Sonso study area of the Budongo Forest Reserve contains a grid trail system consisting of maintained paths that segment the core home range of the Sonso community into ∼100*100 m blocks of forest. This grid system was used for travel by researchers and often chimpanzees. In January 2010, the community consisted of 73 individuals; 11 adult males, 23 adult females, 3 sub-adult males, 11 sub-adult females and 25 juveniles/infants. Adults were defined as individuals above 15 years of age, and sub-adults as individuals between 10 and 15 years and regularly seen without their mothers [57].

### Experimental Design

Focal individuals were exposed to the snake in three different social contexts: when (i) alone and when travelling with others as either the (ii) front or (iii) back individual in the group (minimum of 4 m between focal back individual and individual ahead of them). Seven individuals completed all three of these conditions as part of a within-subjects design, and six additional focal individuals completed single trials. Focal individuals were exposed to the snake at a naturalistic rate (see Supporting Information (SI), File S1).

### Experimental Protocol

#### Snake model

We manufactured a model of an African Rock Python (*Python sebae),* a non-venomous but lethal ambush predator that is common in the Budongo Forest, using the conserved skin of a dead python donated to us by the Uganda Wildlife Education Centre (UWEC). This snake had been brought to UWEC, after it was accidently injured during grass cutting in the local area. Despite treatment, the snake later died as a result of these injuries. Transparent fishing line attached to the head of the python model allowed us to move the model from a distance (see Fig. S1A in File S1 for a picture of the snake model).

#### General trial procedure

**Figure 2:**
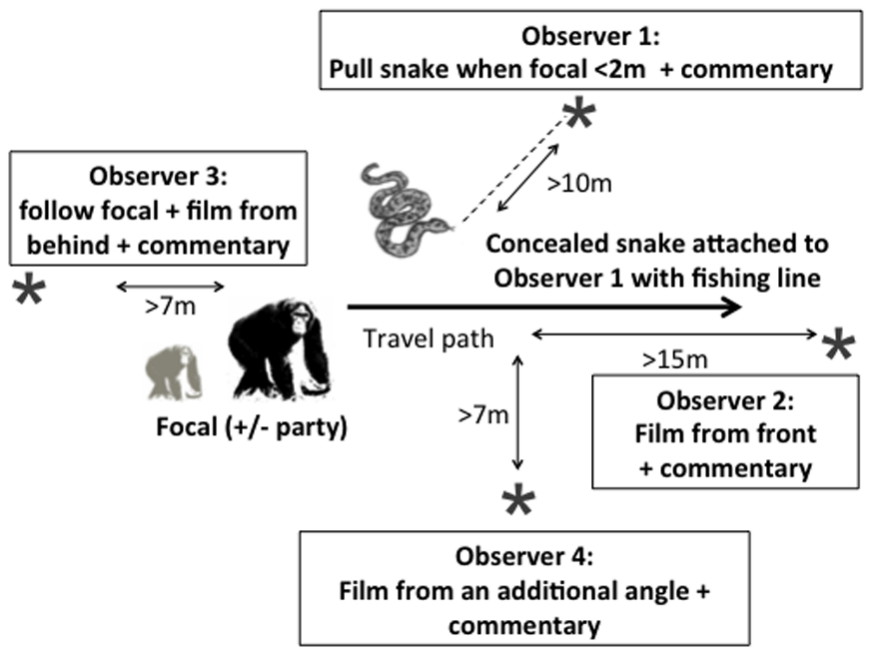
Diagram of the experimental set up. The snake image represents the location of the python model, concealed by leaves not depicted. Observers are depicted by grey asterisks and their main roles defined. Observer 4 was optional in the set-up. The chimpanzee image depicts the focal chimpanzee, who could be accompanied by other group members depending on the experimental condition.

When the focal chimpanzee was travelling on a trail, two observers navigated ahead of this individual and hid the snake model under a pile of local leaves and bark just to side of the focal individual’s anticipated travelling path. Figure 2 shows the relative position and roles of the four observers in an experimental set up. Observers used Motorola GP340 radios to coordinate actions. Once the target focal individual was within a 2 m radius of the snake, Observer 1 pulled once on the fishing line, causing the snake to move about 20 cm and to reveal itself to the chimpanzee. Due to the moving nature of the model, detection of the snake was immediate in all trials, as indicated by head turning towards the snake as it moved and subsequent startle responses of focal individuals. This experimental setup allowed us to reveal the snake to specific focal individuals and to leave the snake concealed if conditions changed and a trial was no longer appropriate. Pulling the snake model from under the leaves meant different amounts of the snake became visible in each trial, avoiding habituation (see Fig. S1B in File S1). Although this also meant the model varied in visual saliency across trials, we found that the percentage of snake model that was visible did not influence the likelihood of non-focal individuals, who saw the static model, producing calls (for further details, see File S1).

### Behaviour Coding

The focal individual was filmed from a minimum of two different angles. After thorough training, all observers gave reliable real time commentary on the looking direction and vocal and anti-predator behaviour (see Table 2) of the focal individual and other group members present, either onto a video recorder or a dictaphone (see File S1 for details on training and inter-observer reliability tests). Observer 2 additionally sound recorded vocalizations with a Sennheiser ME67 microphone and Marantz PCM661 solid state recorder.

### Video Coding

Video recordings were coded (see Table 2) and analysed using the software package *Observer XT 10.0* (Noldus Information Technology, for more details see File S1). Video coding was confirmed as accurate by an independent individual who coded all categories of behaviour in Table 2 on 5/21 within subjects focal trials (25%), resulting in a mean Cohen’s kappa coefficient of.72 (range = .69–.77).

In addition to the behaviours coded in Table 2, in each trial an estimate of the percentage of the snake model uncovered was recorded by AS, and in the majority of trials this was later verified from photographs of the revealed snake model next to a scale object (compass: see Fig. S1B in File S1). We also coded the timing of the arrival of any new individuals who had not been in the focal individual’s party (individuals within 30 m radius of the focal individual; [49]) when the snake was revealed. At the point of an arrival we identified any individual who had seen the moving or static snake (videoed or commentated by observers to have approached and visually fixated on the area containing the snake) and was still in the party as a potential caller, who could produce vocalizations to warn the new arrivals about the presence of the snake. We characterized the relationship between the potential callers and the arriving individuals first in terms of dominance [58] and second in terms of an affiliative relationship or friendship (see File S1 for calculations).

### Calling Behaviour

There is a lack of clarity in how chimpanzee alarm calls have been described and labelled in the literature. Crockford et al. [10] discriminated between loud alarm barks and quiet ‘alert hoos’ but did not provide spectrograms of the alert hoos commonly elicited by their snake model. In this study, we distinguished three types of alarm calls: ‘Soft Huus’ (SH), ‘Alarm Huus’ (AH), and ‘Waa Barks’ (WB; Fig. 1; Audio S1, S2, S3). Although these three call types grade into each other (SH to AH to WB) it is possible to reliably categorise this continuum into three discrete types. To ensure calls were classified correctly, two expert chimpanzee vocal researchers, AS (3 years experience) and KS (10 years experience) independently classified all 1273 focal alarm-related calls that occurred across trials. KS coded the calls blind to the trial type and other behaviour coding. We obtained high agreement on the classification of calls (Cohen’s Kappa = 0.87) and the 78 disagreements all concerned calls from neighbouring classes (SH/AH disagreement = 25; AH/WB disagreement = 53; SH/WB disagreement = 0). KS and AS reviewed all 78 calls where there was disagreement and reached a mutually agreed final classification for these calls, resulting in a final set of 876 SH, 229 AH and 168 WB.

### Observational Data Collection

We conducted focal follows of most adult and sub-adult community members, including all the experimental focal subjects, in order to collect observational data on their social relationships. An average of 190.0 hours (min = 34.5; max = 405.4; SD = 112.9) of focal data were collected on each experimental focal individual over the study period. All occurrence grooming and aggressive interactions involving the focal individual were recorded in addition to 15 minute scans of their nearest neighbour (identity and proximity) and the identity of all individuals within their party (all individuals within a 30 m radius of the focal individual; [49]). These data enabled us to calculate the direction (positive or negative) and magnitude (strong or weak) of social relationships between individuals in the community (for calculations, see File S1). All occurrence pant-grunt vocalizations (call given by subordinate individual to a dominant individual) were collected in order to establish dominance hierarchies [58].

### Operationalisation of Intentionality Criteria

In order to provide a direct comparison to chimpanzee gesture findings, we tested chimpanzee alarm call production against several markers of intentionality typically used in primate gesture research (Table 1). We examined whether alarm calls were (i) used socially by examining sensitivity to the presence or absence of an audience and the composition of the audience; (ii) directed at recipients by examining audience checking and gaze alternation before and during calling; and (iii) goal directed by examining whether callers persisted in vocal production until all group members were safe from danger. We operationalised these criteria to maximize the similarity with previous gesture work as detailed below.

#### Social use: presence/absence of an audience

In line with previous ape gesture studies (e.g. [26], [27]) that examined signalling in the presence and absence of an audience in order to establish that the signals are communicative, we compared focal calling behaviour in the alone and social group conditions. For this, we used the 21 focal within subject trials (N = 7 individuals, N = 3 trials (1 alone; 2 social) per individual). However, in three of the seven focal alone trials, other individuals called or approached the focal individual after the snake was revealed, providing the focal individual with an audience and converting the trial into a social trial. Thus, in order to compare vocal behaviour across the seven individuals who completed both the alone and social conditions, we had to examine the focal individual’s immediate response to the snake. FK alone, MS alone, and RE alone trials became social trials 8.24, 9.56 s and 36.76 s (mean 18.19 s) respectively after the focal animal saw the moving snake. Thus, as the shortest alone trial was 8.24 seconds, we focused the analyses on the immediate response of the focal individual in the first 8.24 s of all within-subjects trials, to determine if focal individuals took into account the presence or absence of an audience when producing their calls.

#### Social use: composition of audience

In order to determine if chimpanzee alarm calls were mediated not only by the presence of an audience but also the identity of individuals present, we examined whether the arrival of dominant or important social partners influenced the calling behaviour of both focal and non-focal individuals who had seen either the moving or non-moving snake model. For this, we examined all 27 trials and identified all instances where the audience composition changed due to new individuals joining the focal party after the snake had been revealed (i.e. individuals who had not seen the snake and may or may not have heard alarm calls before joining the party). We examined the calling behaviour of all individuals who had seen the snake in the 30 seconds before and after the arrival of new individuals. We assessed whether call rates increased in response to arrivals and if so whether the identity of the individual arriving mediated the change in calling behaviour. This would indicate that the calls were not only communicative but may have been directed at specific members of the audience. As an alternative explanation we also examined factors that were related to the caller’s own danger levels and proximity to the snake, which may indicate calls were an individualistic expression of emotion (zero order intentionality) rather than signals directed at others.

#### Social trials

The remaining intentionality criteria (audience checking, gaze alternation and persistence) all require a social audience. For the analyses that follow we thus examined the behaviour of the focal individual in the 22 trials where a social audience was present at some stage (5 alone trials where no other individuals arrived were excluded). For 19 trials where a social audience was present from the moment of snake discovery, the whole experimental trial was considered, but as MS, FK and RE alone trials only became social (other individuals arriving or because of out of sight individuals calling) at 9.56 s, 8.24 s and 36.76 sec after the snake was discovered, for these trials we only considered the focal animal’s behaviour after these periods once a social audience was present.

#### Audience checking

In line with Hobaiter and Byrne [23] we examined whether the signaller showed awareness of potential recipients and looked at the recipient(s) before signalling. We examined whether, in the 5 sec period before producing a new call bout, the focal individual looked at the snake (0 = no; 1 = yes), at another visible chimpanzee in the party (0/1), or at any visible or out of sight, but calling individual (0/1). It was important to consider individuals out of sight, because vocalizations, in contrast to visual gestures, can be directed to any member of the auditory audience (i.e. all those in hearing range of the call), regardless of whether they are visible or not.

#### Gaze alternation

For all social trials, we identified whether gaze alternation between a social partner and the snake occurred during the focal individual’s production of snake alarm calls. For this, we followed Leavens et al. [27] and Leavens and Hopkins [28] and identified gaze alternations (i.e. the focal individual looks successively from snake to chimpanzee or vice versa) that did and did not accompany calling behaviour. Gaze alternation is a measure that identifies whether the signaller checks the behaviour and attentional state of its recipient when communicating [28], and may even be used to communicate about an external entity [28]. As many chimpanzees approached the snake silently, but engaged in behaviours that indicated awareness of the danger (e.g. bipedal stance, visual scanning of the snake area), attending to the behaviour of other chimpanzees through visual checking and gaze alternation is likely important for callers. Therefore, if calling was mediated by the behaviour of others and others’ awareness of the danger, we would expect to see gaze alternation accompanying calling.

Leavens et al. [27] identified cases of gaze alternation that occurred during pointing gestures (mean duration 4.9 s) as a marker of intentionality. However, as alarm calls were considerably shorter in duration than pointing gestures, we could not examine gaze alternations that occurred within these signals. Instead, we looked for gaze alternation events where calls occurred within 3 seconds of the change in gaze focus (e.g. within a 6 sec period surrounding the switch in visual gaze between the snake and social partner). All gaze alternation events were thus categorized as gaze alternations with calls, or gaze alternations without calls.

To determine if gaze alternations were communicative we calculated rates of gaze alternations with calls (Gaze alternations with calls/duration focal calling bouts in trial) and without calls (Gaze alternations without calls/duration of trial without focal calling bouts). For each individual we assessed if their rate of gaze alternation was higher with or without calls.

To determine whether gaze alternations were produced more often with certain call types, we first determined the type of call associated with each gaze alternation with calls (N = 76) using standardized rules (see File S1). We then determined for all call bouts whether or not the focal individual produced at least one gaze alternation event associated with the relevant call type during the bout (e.g. for each SH bout we looked for the occurrence of at least one gaze alternation associated with SH).

#### Persistence: goal directed behaviour

In line with previous gesture studies [19], [30], we took the repetition of signals until a goal is met as a marker of intentionality and goal-directed behaviour. Identifying the conditions that are associated with the cessation of signalling is important in order to infer the goal of a signal sequence. Within calling bouts, chimpanzee alarm calls are commonly repeated in sequences, with our data revealing a mean duration of 2.49 s (SD = 3.4 s) between individual calls, which is comparable to repetition rates observed in gesture sequences [32]. We hypothesized that the goal of alarm calls was to warn others of danger and we therefore tested whether focal chimpanzees persisted in producing alarm calls until all others in the vicinity of the snake were safe (either due to sufficient physical distance [>10 m away, up a tree] or aware of this ambush predator - [produced calls, approached bipedally or visually scanned the area containing the snake]; See Table 2). More specifically, we examined if others in the vicinity were more likely to be safe in the 10 s after the last call in a bout was produced compared to the rest of the trial (defined as period from snake exposure to when the focal individual moved more than 10 m from the snake), excluding the 10 s periods associated with the stopping of calling. We took behaviour in the rest of the trial period to be indicative of a ‘chance’ level of a particular behaviour occurring in this predatory context. The end of a calling bout was defined by the last call before a period of at least 30 sec silence. In order to test an alternative hypothesis that alarm calls were a reflection of the perceived level of threat to the caller, rather than a recipient-directed signal, we also tested whether the caller was more likely to have started moving away from the snake and thus reduced his own risk level as he stopped calling, compared to the rest of the trial.

### Statistics

Generalized Linear Mixed effects Models (GLMMs) with a binomial error structure were used to investigate the influence of continuous or categorical explanatory variables (e.g. distance to snake, higher ranking individual present) on a binary response variable (e.g. call or not). Furthermore, because we had repeated sampling from the same individual due to both multiple calling bouts within an experiment and multiple experiments on some individuals we fitted “individual” and “experiment” as random factors [59] by conducting random intercepts models using the package lme4 [60]. When investigating the influence of multiple fixed explanatory factors we used Akaike’s Information Criterion (AIC) to select the most parsimonious model. Lower AIC values indicate improved support for each model [61], [62] with terms considered to improve the fit only if their exclusion from the model inflated the AIC value by more than two units [63]. To assess the significance of explanatory variables, we returned each variable one at a time and compared this to a null model, comprising only the intercept and random effects, using a likelihood ratio test [64]. Models were implemented in R v. 2.12 and alpha values were set at 0.05.

In addition, we performed simple within-subjects non-parametric tests to establish if calling behaviour varied across the three different conditions that seven focal chimpanzees experienced. We also conducted One sample Wilcoxon signed Ranks tests (non-parametric equivalent to one-sample t-test used due to skew in data set) to establish if the behaviour observed in the 10 second period associated with stopping calling (e.g. 10 s after the last call in a bout) was significantly different from behaviour in the rest of the trial (chance level of that behaviour occurring). To avoid pseudo-replication, for each individual we calculated mean values for the behaviours of interest in the two time periods across all their social trials. These statistical tests were conducted using PASW 18 software and all tests were 2-tailed.

## Results

### Social Use: Presence/Absence of an Audience

We investigated whether calls were directed at recipients and therefore only produced in the presence of an audience, by comparing the calling behaviour of seven focal individuals who had each discovered the moving snake in one alone and two social contexts (N = 21 trials). We found that only soft huus (SH) were produced as an immediate response (within the first 8.24 s) to snake discovery in a sufficient number of trials (16/21) to enable statistical analysis. For this call type, our results showed that calls were produced irrespective of the presence of an audience, indicating these calls were not directed at conspecifics. More specifically, in the first 8.24 s after snake exposure, six of the seven individuals produced SH in at least one of the social conditions (6/7 in front; 5/7 in back) and five of the seven individuals also produced SH in the alone condition. A Friedman test revealed no difference in the number of SH given in this time period across the three trial types (X^2^(2) = 1.52, p = .531). A very similar pattern was found when considering the focal animals’ responses over a longer time period (see File S1). An additional two chimpanzees completed alone trials, and both of these individuals produced SH within 8.24 s of encountering the snake, confirming the pattern that SH are produced in both the presence and absence of an audience. In immediate response to the snake (within 8.24 s), AH were produced in only one alone condition and WB were produced in only one social (back) condition.

When taking into account the complete duration of the within subject trials, AH were produced by focal individuals in 9/21 trials, after an average latency of 61.86 s (range 6.76–170.76 s, SD = 63.73) from snake discovery. WB were produced by focal individuals in 7/21 trials, after an average latency of 52.94 s (range 5.52–123.82, S.D. = 49.06) from snake discovery. In contrast, when taking into account the complete duration of the trials, SH were produced in 20/21 trials, after an average latency of 7.60 s (range 1.77–50.36 s, SD = 9.85) from snake discovery (see Video S1).

### Social Use: Composition of Audience

To assess if call production was mediated by the composition of the audience, we identified instances where the audience composition changed due to new individuals joining the focal party after the snake had been revealed. For this, we considered data from focal and non-focal individuals from all 27 trials and found we could accurately determine the timing of 39 arrival events across 9 trials, where a new individual joined the focal party. These individuals arrived on average 9.88 minutes (SD = 8.68 min) after the snake was revealed (range = 1.05–28.98 min). Across trials, we identified 14 different individuals who were in a position to act as callers in one or more arrival events: these were individuals who had seen the snake before the new individuals arrived and therefore had the opportunity to increase their calling in response to a newly arriving individual. In total, we identified 87 dyads of potential callers and newly arriving individuals. For each potential caller in a dyad, we determined whether or not their SH, AH and WB production increased in the 30 seconds after an arrival compared with 30 seconds prior to an arrival.

Potential callers increased their SH production in 2/87 dyads in the 30 seconds after a new individual arrived in the party. This implies that SH production is not mediated by the arrival of new individuals and there was insufficient variability in the data to statistically investigate this further. In contrast, potential callers increased both AH and WB production in 9/87 dyads in the 30 s after new individuals arrived compared to the 30 s before they arrived. There was sufficient variability in these data to investigate whether an increase in both AH and WB production was mediated by social factors, such as the identity of the newly arriving individual, or rather by the potential caller’s own position relative to the snake. A GLMM with a binomial error structure was implemented, with increase in both AH and WB production (0/1) as the dependent binary variable and individual and experiment entered as random factors. As independent variables, we included an index value for the presumed ‘friendship’ between the potential caller and the newly arriving individual (see File S1), their dominance relation (see File S1), the potential caller’s absolute distance to the snake, and whether he or she was closest to the snake relative to others. The GLMM revealed that an increase in AH and WB production was significantly mediated by the friendship between the potential caller and the arriving individual, with the arrival of friends more likely to be associated with an increase in calling (X^2^ (1) = 9.68; p = 0.002). In contrast, although potential callers appeared more likely to increase AH and WB production to the arrival of an individual who was dominant to them, this trend was not significant (X^2^ (1) = 2.89; p = 0.089). In terms of the potential callers’ own position relative to the snake we found that there was a non significant trend for individuals to increase AH and WB production in response to others arriving if the callers were the closest individual to the snake (X^2^ (1) = −2.92, p = 0.088), however, callers’ absolute proximity to the snake did not influence their AH and WB production (X^2^ (1) = 0.01, p = 0.923).

### Audience Checking

**Figure 3:**
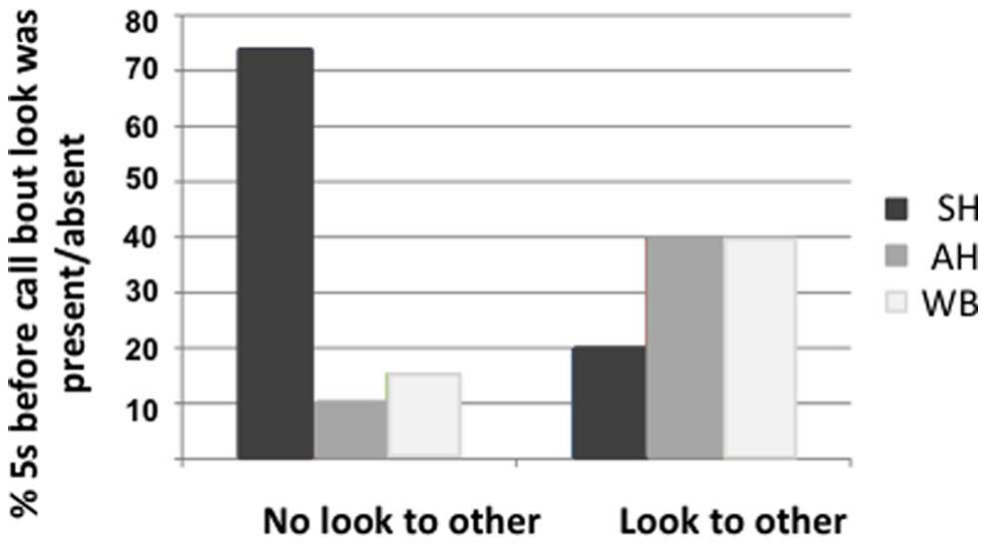
Percentage of cases (raw data) where a look to another chimpanzee was present/absent in the 5 sec before a new call bout as a function of the type of call then given.

To further assess if chimpanzees were aware of their audience and directed alarm calls at others, we examined the focal animal’s gaze orientation in the 5 seconds before starting a new call bout (see Table 2 for definition). For this, we only used focal data from trials or parts of trials where a social audience (visible or in auditory contact) was present (total of 22 trials from 12 focal individuals). We predicted that, if calls were directed towards others, the caller should look to the intended recipient(s) prior to starting a call bout. We coded whether, in the 5 s period before a new call bout, the focal individual looked at the snake (0/1), at another visible chimpanzee in the party (0/1), or at any visible or out of sight, but calling individual (0/1). We ran three separate GLMM’s with binomial error structures to investigate whether the variation in gaze orientation at the three targets 5 s before the start of a call bout could be explained by the call type following it (SH, AH, WB). We identified a total of 49 cases from 12 individuals over 22 trials where we could accurately code gaze orientation 5 s before the start of the new calling bouts. We found a non significant trend for chimpanzees to be more likely to look at the snake before making a SH than an AH or WB (X^2^ (2) = 5.46; p = 0.065). In contrast, we found that call type explained a significant amount of variation in gaze orientation towards other chimpanzees (X^2^ (2) = 10.21; p = 0.006): chimpanzees were more likely to look at another chimpanzee in the 5 s before producing AH or WB compared to SH bouts (see Fig. 3). As receivers of vocal signals can be out of sight, we also examined cases where chimpanzees oriented towards the location of non-visible individuals (identified by their calls). In line with the previous result, we found that call type explained a significant amount of the variation regarding whether the focal individual oriented his attention towards visible or auditory conspecific targets 5 s before starting a call bout (X^2^ (2) = 15.42; p<0.001). Again the focal individual was more likely to attend to social stimuli before producing an AH or WB bout than a SH bout. Video S1, Video S2 and Figure S2 in File S1 together illustrate an example trial where an adult female looked at the snake before and during production of a SH bout, but looked at another individual before beginning a WB bout.

### Gaze Alternation

As a further marker of calls being directed at recipients and callers monitoring the behaviour of others, we examined whether chimpanzees accompanied their vocal signals with gaze alternation between recipients and the snake. For this, we only used focal data from trials or parts of trials where a visible social audience was present (total of 21 trials from 12 focal individuals). Nine out of twelve focal individuals (75%) who had seen the moving snake showed gaze alternation with calling (see Video S2). Eight of these nine chimpanzees alternated their gaze between the snake and another chimpanzee at a significantly higher rate during calling bouts (median = .029 gaze alternations/s; IQR = .035) than when they were not calling (median = .006 gaze alternations/s; IQR = .020; two-tailed sign test, p = .020).

Overall, gaze alternation was associated with 15/38 (39%) SH bouts, 3/13 (23%) AH bouts and 4/11 (36%) WB bouts. A GLMM confirmed that call bout type did not explain a significant amount of variation in the occurrence of gaze alternation (X^2^ (2) = 1.24, p = 0.538).

### Persistence: Is Signalling Goal Directed?

In order to identify the ‘stopping rules’ for call production and to test the hypothesis that individuals persist in calling until their goal of warning others about danger is met, we compared the behaviour of callers and recipients in the 10 s after the last call in a calling bout (stopped calling) with behaviour during the rest of the trial. For this, we used focal data from trials or parts of trials where a visible social audience was present, and where the focal individuals were still present within 10 m of the snake when they stopped their calling bouts (total of 19 trials from 11 focal individuals). A one sample Wilcoxon signed rank test revealed that a decrease in the caller’s own risk did not explain their decision to stop calling, as callers were not more likely to be moving away from the snake as they stopped calling than in the rest of the trial (z = 1.07, N = 11, p = .284). Rather, it appeared that a decrease in other individuals’ risk explained the callers’ decisions to stop calling: when callers stopped calling, it was significantly more likely that all recipients were safe (aware of snake, more than 10 m away from it, or up in a tree; Table 2) compared to chance (median = 100.00% vs. 40.42%; z = 2.54, N = 11, p = .011). These analyses indicate that the goal of calling could be to warn others of danger, as it is a decrease in the risk of others, not the caller, which seems to mediate the decision to stop calling.

## Discussion

The results of our study demonstrate that some chimpanzee alarm calls show numerous hallmarks of intentional communication. Similar to chimpanzee gestures, WB and AH were produced socially in the presence of socially important individuals: chimpanzees who had seen the snake were more likely to increase their production of WB and AH to the arrival of a friend than a non-friend and there was a trend for increased calling when a more dominant individual arrived. Thus chimpanzees seem to produce these calls tactically and target important individuals who are valuable to them. This is in line with two previous studies, reporting that chimpanzees were more likely to produce alert hoos in the presence of social bond partners [10] and more likely to produce food-associated calls when a dominant friend arrived in the vicinity of their feeding tree compared to other types of individuals [52]. Similar to Crockford et al. [10], we found little evidence that calling was a reflection of the caller’s own risk (zero-order intentionality), as calling was not related to the callers’ absolute distance from the snake. Instead, the production of AH and WB calls seemed to be recipient focussed. Interestingly, there was a trend for chimpanzees to increase alarm calling if they were the closest individual to the snake when a new individual arrived. Again, this finding is in line with Crockford et al. [10], indicating that the individual closest to the danger may perform a form of sentinel duty. In addition, our study is the first to demonstrate that the production of WB and AH calls was often preceded by visual checking of the audience, accompanied with gaze alternations, and individuals were likely to persist in producing calls until all group members were safe from the ambush predator.

We interpret these patterns as evidence that chimpanzee alarm calling meets the key diagnostic features of intentional signal production. Although each of these behaviours can be explained separately as the product of less complex cognitive processes, the combined overall pattern is more consistent with the hypothesis that call production is both socially directed and goal-directed. Furthermore, following previous gesture work where the production of manual signals in concurrence with these markers of intentionality has been taken as evidence for (first order) intentional communication, we suggest that these two chimpanzee alarm call types qualify as intentional signals.

The debate as to how well behavioural markers can distinguish between first-order and zero-order intentionality in relation to signalling will continue. However, a key point of our results is that chimpanzee vocalizations meet the same basic criteria for intentional signal usage which have been put forward for great ape and human infant gestures. At the same time, our results are inconsistent with the traditional notion of primate vocalizations being reflexively and unintentionally produced. We thus argue that our results undermine a key line of argument for gestural theories of language evolution and highlight the need for care in interpreting and comparing primate communication data from different communicative modalities [44]. In contrast to gestural research that has mainly focused on great apes, in the vocal domain monkey species have been the predominant subject of research [44] and studies have indicated that some of their calls are not directed at others, but are instead a reflection of the internal state of the caller [6], [65] and indicative of zero order intentionality. Proponents of gestural theories of language evolution have commonly taken these findings as representative of primate vocal communication as a whole, but our results show that generalizing findings from monkey species to great apes may not be valid. This study complements a growing body of evidence showing similarities in the proximate mechanisms driving the production of both great ape vocalisations and gestures and indicating that both modalities are characterised by voluntary control and flexible, intentional use of signals [19]–[36]. This indicates that rather than having an exclusively gestural or vocal origin, language may have originated from a multimodal communication system, containing both gestural and vocal signals [18], [55].

In contrast to the WB and AH, we found mixed support for SH being produced intentionally. SH tended to be given in immediate response to the discovery of the moving snake model, regardless of whether or not there was a conspecific audience. In addition, SH production very rarely increased in response to the arrival of new individuals and callers were significantly less likely to monitor or check the audience before starting a SH calling bout than an AH or WB bout. Thus, at least in immediate responses to a moving snake, SH calls do not seem to be directed at others in an intentional way and may thus be interpreted as the product of zero-order intentionality. SH did, however, still meet some of the markers of intentional signalling as they were accompanied by gaze alternation and they contributed to the finding that individuals were likely to persist in calling until all group members were safe. One possible explanation for this mixed pattern of results is that initially upon discovery of a predator, these calls are part of a relatively involuntary startle response and may represent an individualistic expression of fear towards a salient moving snake model [66], [67]. It is possible, however, that the same calls are used in a more recipient directed and intentional manner later in the trial, after the initial startle response and peak of arousal associated with discovery of the predator has dissipated. In contrast to Crockford et al. [10] we used a moving snake model manufactured from real python skin, rather than a static papier-mâché model, suggesting that we triggered more powerful startle responses, which may have required longer recovery times before individuals regained voluntary control over their call production.

One outstanding issue concerns the motivation underlying WB and AH production. It is possible that chimpanzees have a selfish motivation and call to recruit others in order to obtain support or comfort in a stressful and dangerous situation. If this is the case, then these vocalizations are functioning in a similar manner to many great ape gestures in that they are an imperative request for action in others [20], [68]. However, given the context-specific nature of many primate alarm calls [69]–[71] these signals could also be produced with an intention to benefit others by providing information about a specific danger. Indeed, our finding that calling is more likely to stop when all individuals are safe indicates that the goal of callers may be to warn others of the danger; a potential case of intentional referential communication.

In conclusion, chimpanzee alarm call production meets some of the key hallmarks of intentional signal production in a directly comparable way to chimpanzee gestures. We have shown that AH and WB production is sensitive to the composition of the audience and that these calls are directed at specific individuals and goal-directed. We therefore conclude that some chimpanzee vocal signals qualify as intentional signals, in contrast to the traditional characterisation of great ape vocal behaviour as an inflexible, reflexive, involuntary, zero-order intentional process [12]. We believe that our findings are inconsistent with central arguments of gestural theories of language origins and instead support a multimodal origin for human language.

## Supporting Information

File S1 **Supplementary information including supplementary methods, results, figures, video legends and audio file legends.**
(DOC)

Video S1 **Video** **illustrating discovery of the moving snake and soft huu production.** Video is filmed from position 1 illustrated in Figure S2 in File S1. Focal adult female, Nambi produces a startle response and then begins producing soft huus (commentated by the observer as ‘huuing’) and visually examining the snake, from a bipedal stance. Nambi’s gaze remains fixated on the snake during the soft huu production.
(AVI)

Video S2 **Video illustrating gaze alternation and looking at a group member before producing waa barks.** Video is filmed from position 2 illustrated in Figure S2 in File S1. Focal adult female Nambi reacts to the arrival of her adult son Musa by turning and looking at Musa before producing her first waa barks of the trial. Nambi then looks immediately back at the snake, showing gaze alternation between the recipient and the snake whilst calling. During Nambi’s waa bark production, Musa stands bipedally.
(AVI)

Audio S1 **Sound recording of an example Soft Hoo (SH).**
(WAV)

Audio S2 **Sound recording of an example Alarm Hoo (AH).**
(WAV)

Audio S3 **Sound recording of an example Waa Bark (WB).**
(WAV)
