# Supplementary material for: Chimpanzee Alarm Call Production Meets Key Criteria for Intentionality
Source: PLoS One. 2013 Oct 16;8(10):e76674. doi: 10.1371/journal.pone.0076674 (PMC3797826; doi:10.1371/journal.pone.0076674)
Supplement: File S1 — Supplementary information including supplementary methods, results, figures, video legends and audio file legends. (DOC) [file pone.0076674.s001.doc]

**FILE S1: SUPPORTING INFORMATION**

***Experimental design***

The focal individual was exposed to the snake in three different social contexts: (1) when alone: no other individual within 50m of the focal; and (2) when travelling with others in a social group (other individuals within 30m), either as (i) the lead individual at the front of a

travelling party or (ii) as the last individual at the back of a travelling party. In order to reveal the snake in the back condition, there had to be at least 4 meters between the focal and the individual ahead of them. Seven individuals completed all three of these conditions in a counterbalanced order: these were four adult males, two subadult males and one subadult female. In addition to these seven animals, two adult females, three adult males and one subadult female were the focal animals in single experiments.

*General trial procedure: Snake exposure*

For the seven focal individuals with repeated exposures to the moving snake, the mean inter-trial duration was 68 days (SD = 49; Range = 15-142 days). This reflected naturalistic chimpanzee-snake encounters, as on average, we observed a snake (i.e. any type of dangerous snake, such as Jameson’s mamba, gabon viper, puff adder, black cobra, spitting cobra, or python) while following the chimpanzees approximately once a month, depending on the area the chimpanzees were ranging in and the season. A further 16 individuals saw the non-moving snake once it had been revealed to the focal and for individuals who saw the snake in multiple trials, on average there were 100 days (SD = 116; range 4-265 days) between exposures.

***Behaviour coding***

As pilot work indicated that soft hoos (SH) could be difficult to detect from videos and exact gaze direction was difficult to ascertain on 2D video images, all observers were thoroughly trained to commentate on the looking direction and calling behaviour of the focal. We assessed the accuracy and consistency of commentary, before the experimental trials began, in a test period where all observers simultaneously commentated the looking behaviour of a focal chimpanzee in relation to a specified object (standing in for the snake). We coded the number of looks to the ‘snake’, to other chimpanzees and elsewhere in the test period, and calculated the duration of each of these looks. High levels of inter-observer reliability were obtained between the four observers with a mean Cohen’s Kappa score of 0.88 (SD = 0.14) across observers for the categorical data on frequency of looks at each of the three targets and a mean Spearman’s Rho coefficient of .97 (SD = .03) across observers for the continuous data on duration of agreed looks.

***Video Coding***

The Observer 10.0 software allowed us to analyze and code two video clips from different observational angles simultaneously and to continuously code the focal’s looking and vocal behaviour as well as its movement patterns and the relevant behaviour of others in the party (see Table 2). When needed, it was possible to analyze video clips frame-by-frame. Focal vocalizations and commentary on the arrivals and approaches of others recorded on the dictaphones or sound equipment were extracted manually from their commentary and added to the Observer coding.

***Determination of rank***

When new individuals arrived in the focal party we determined whether the ‘arriver’ was higher ranking than the potential caller. Among chimpanzees, all adult males are dominant to adult females, so when the potential caller was an adult male, we classified all female arrivers as lower ranking. For sub-adult males who were potential callers, all the adult females who arrived in the party during the experiment had been observed giving pant grunts (a vocal signal of subordinancy) to these potential callers during the study period, therefore we assumed that the females were lower ranking than these sub-adult males. Among adult males, chimpanzees form linear dominance hierarchies and we determined ordinal dominance ranks for all individuals using data on the direction of pant grunts. This enabled us to code whether a male arriver was dominant to a male potential caller. As male ranks can change dynamically with time, we calculated male ranks every 3 months during the study period. The analysis was carried out using the Matman Software Package (version 1.1, Noldus Information Technology, [1]) and all male dominance hierarchies were significantly linear using a two-step randomization procedure with 10,000 iterations [2].

When an adult female was the potential caller, all male arrivers were categorized as higher ranking. Unfortunately we had insufficient pant grunt data from female-female interactions to accurately calculate linear ranks for them, so for the 16 cases where a female potential caller had a female individual arrive into the party, we were conservative and coded that the arriving individual was not higher ranking than the potential caller.

***Calculation of friendship indices***

We calculated a composite friendship index for all potential callers. All potential callers had been the subject of at least 12 hours of focal observation between September 2010-December 2011 and we calculated an index value between each potential caller and 33 other adult or subadult members of the community who were regularly observed (total 33 dyads). The index was modified from Gilby and Wrangham’s [3] composite association index and used measures of party-level association (PLA), nearest neighbour association and grooming frequency. For party-level association, we used the Simple Ratio Index (4) to assess the proportion of time that a dyad was observed in the same party together. The PLA for chimpanzees A and B is calculated using the 15-min scan data on party composition and would be:


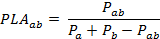


where P_ab_ is the number of parties containing A and B together, P_a_ is the number of parties containing A, and P_b_ is the number of parties containing B. In the denominator, P_ab_ is subtracted to avoid counting individuals twice since the P_a_ and P_b_ counts already include parties where individuals A and B are seen together.

We also calculated a nearest neighbour (NN) index, a measure of spatial proximity, which was the rate that an individual was the nearest neighbour (within 10m of the focal) to the focal. The spatial proximity between focal individual A and chimpanzee B is calculated using the following formula:


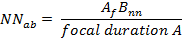


where A_f_B_nn_ is equal to the number of 15 scans where A is the focal and B is the nearest neighbor within 10m of A. The denominator is the duration of the focal observation time of individual A.

Finally, we calculated a measure of behavioural association by calculating grooming rates between all potential callers and the other regularly observed members of the community. Using the all-occurrence grooming records of the focal individuals, the grooming rate between chimpanzees A and B was calculated as follows:


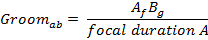


Where A_f_B_g_ is the duration of grooming between individuals A and B when A was the subject of a focal observation and the denominator is the duration of the focal observation time of A.

To calculate the composite friendship index (CFI) we took the mean of these three indices. We then identified strong bonds in the community, but we were specifically interested in how potential callers valued their relationships with individuals arriving in parties. Therefore, for each potential caller, we calculated a mean CFI value as well as the standard deviation of these values. We then used standard z-scores to capture the direction and magnitude of the relationship between the potential caller and the arriver, in relation to the potential caller’s other dyadic relationships. The calculated z-score between potential caller A and arriver B is:


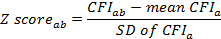


where CFI_ab_ is the composite friendship index between the two individuals, the *mean CFI_a_* is the average of all dyadic CFI values between the potential caller and all partners in the community and the denominator is the standard deviation of these values. This method allows us to control for an individual’s general tendency to associate with others. For example, a gregarious potential caller might have high CFI scores with everyone. By considering each individual caller we can identify individuals with whom he/she shares a strong bond relative to all of his/her other relationships.

***Gaze alternation***

In order to determine whether gaze alternations were produced more often with certain call types, we first determined the type of call associated with each gaze alternation event (N = 76). In 56 cases, there was only a single type of call present in the 6 second period surrounding the gaze switch. In 20 cases where multiple call types occurred in this gaze alternation period we applied standardized rules to attribute the gaze alternation to a single call type. We first classified it according to the call type that occurred most frequently during this time period (N = 16 cases). If there was no call type that was more frequent than the rest (e.g. equal numbers of 2+ call types), we classified the event according to the call type that occurred while the caller was looking at the social partner (N = 3 cases). If there was an equal number of different call types produced while looking at the social partner, we classified the GA event according to the call type that was produced closest to the gaze switch from snake to the social partner (N = 1 case).

***Presence / absence of audience: analysis over a longer time window***

In order to compare vocal behaviour across the seven individuals who completed both alone and social conditions, we focused on examining the focal’s immediate response to the snake (8.24s). Here, we assessed if a similar pattern of calling behaviour was maintained over a slightly longer period than the immediate first 8.24s after snake exposure. Because FK alone, MS alone, and RE alone trials became social trials 8.24, 9.56s and 36.76s (mean 18.19s) respectively after the snake was revealed, we examined the first 8.24, 9.56 and 36.76s of FK, MS and RE’s alone trials respectively and the first 18.19s of all other trials. This ensured that overall we examined calling over an equal duration of alone and social trials. Results showed that all seven focal individuals produced SH in at least one of the group conditions (7/7/ in front; 5/7 in back) and 6/7 individuals also produced SH in the alone condition. A Friedman test revealed no difference in the number of SH given in this time period across the three trial types (X^2^(2) = 1.56, p = .502).

***Control analysis: Did the variation in the amount of the snake model revealed affect calling?***

The percentage of the model snake revealed on any given trial varied from 15-97.5% (N=27 trials). We tested whether calling behaviour was likely to have been influenced by the saliency of the stimulus presented by examining whether the percentage of the model revealed affected the decision of non-focal individuals to call. We focused on non-focal individuals who saw the static snake, as focal individuals, who saw the moving snake, called in 26/27 trials. We identified a total of N= 39 cases over 14 trials, when one of 16 non-focal individuals saw the non-moving snake and there was no uncertainty as to whether they called or not. A GLMM with a binomial error structure and individual and trial as random factors revealed that the percentage of snake revealed did not explain a significant amount of variation in non focal calling behaviour (z = 1.51, p = 0.131). This suggests that although there was variation in the saliency of the stimulus presented in each trial, this does not seem to have affected the basic decision to produce calls.

***References***

1. Vries H de (1995) An improved test of linearity in dominance hierarchies containing

unknown or tied relationships. Anim Behav 50: 1375-1389.

2. Vries H de, Netto WJ, Hanegraaf PLH (1993) Matman: a program for the analysis of

sociometric matrices and behavioural transition matrices. Behaviour 125: 157-175.

3.Gilby IC, Wrangham RW (2008) Association patterns among wild chimpanzees (Pan

troglodytes schweinfurthii) reflect sex differences in cooperation. Behav Ecol Sociobiol 62:

1831-1842.

4. Cairns SJ, Schwager SJ (1987) A Comparison of Association Indexes. Anim Behav

35: 1454-1469.

***Figures***


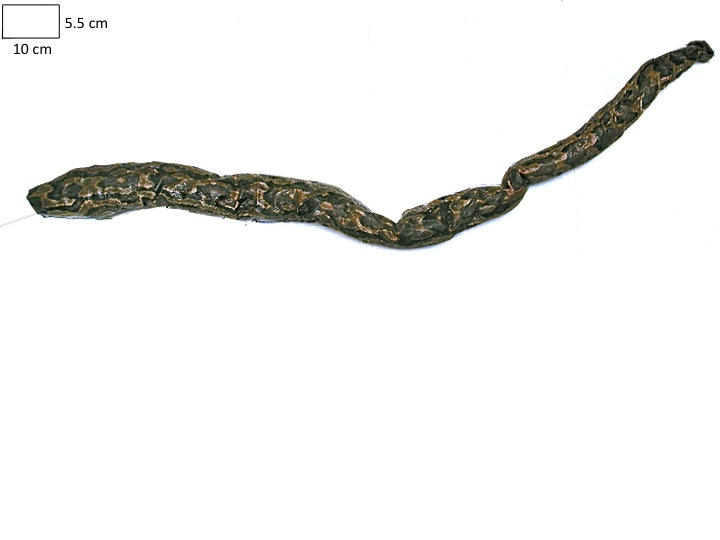


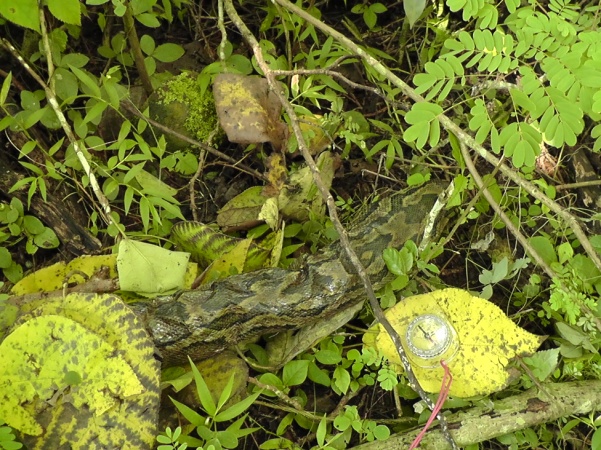

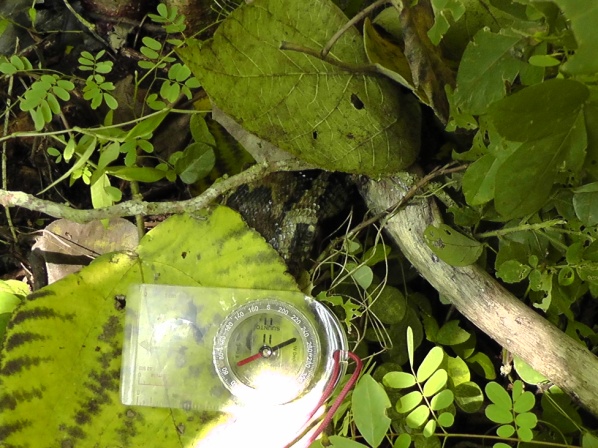


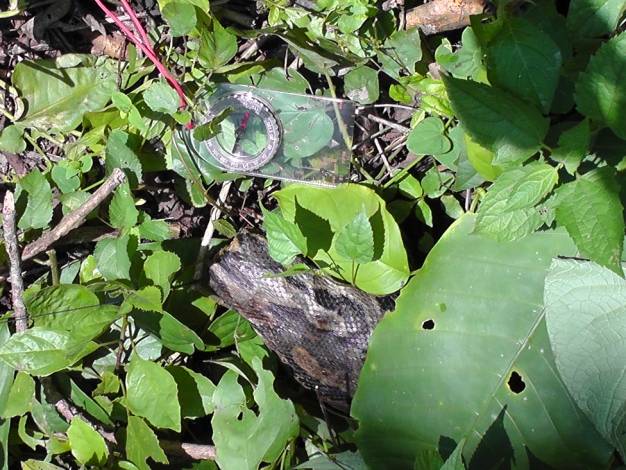

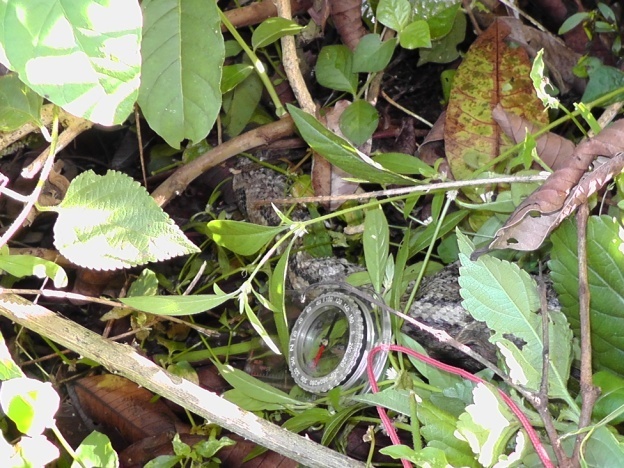


**Figure S1 Photos of the snake model. (A)** Complete snake model next to scale. **(B)** Different areas of snake model exposed to focal chimpanzees after uncovering the model by pulling at the fishing line (attached to the head). Scale item (compass) measures 10*5.5cm.


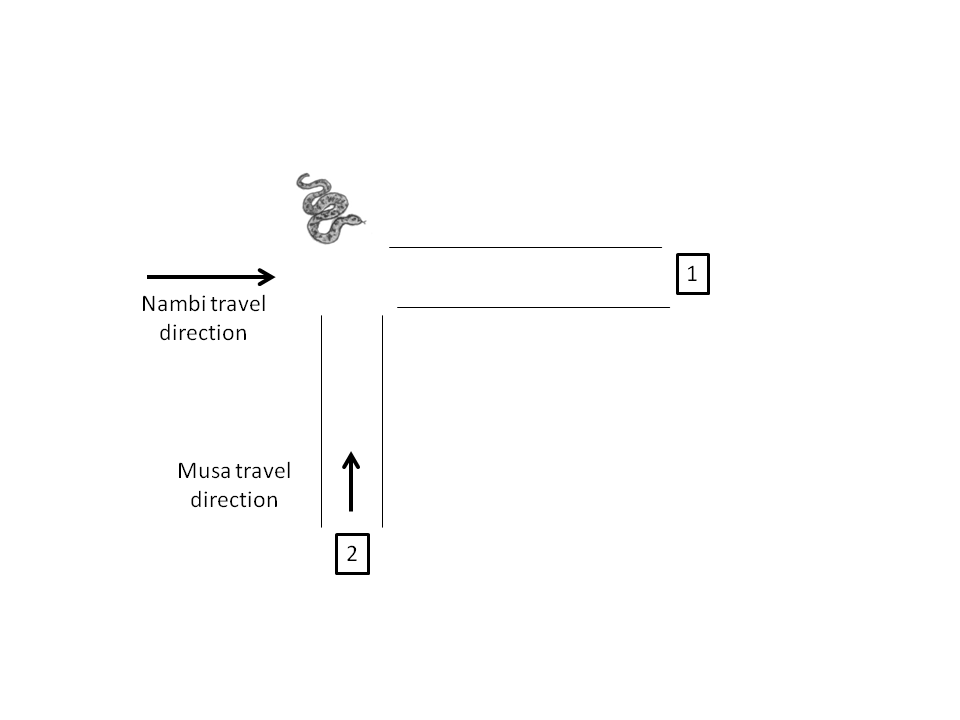


**Figure S2**. Diagram showing the perspective from which Video S1 and S2 were filmed during Nambi’s trial. Videos S1 was filmed from location 1 and Video S2 was filmed from location 2.

***Videos***

**
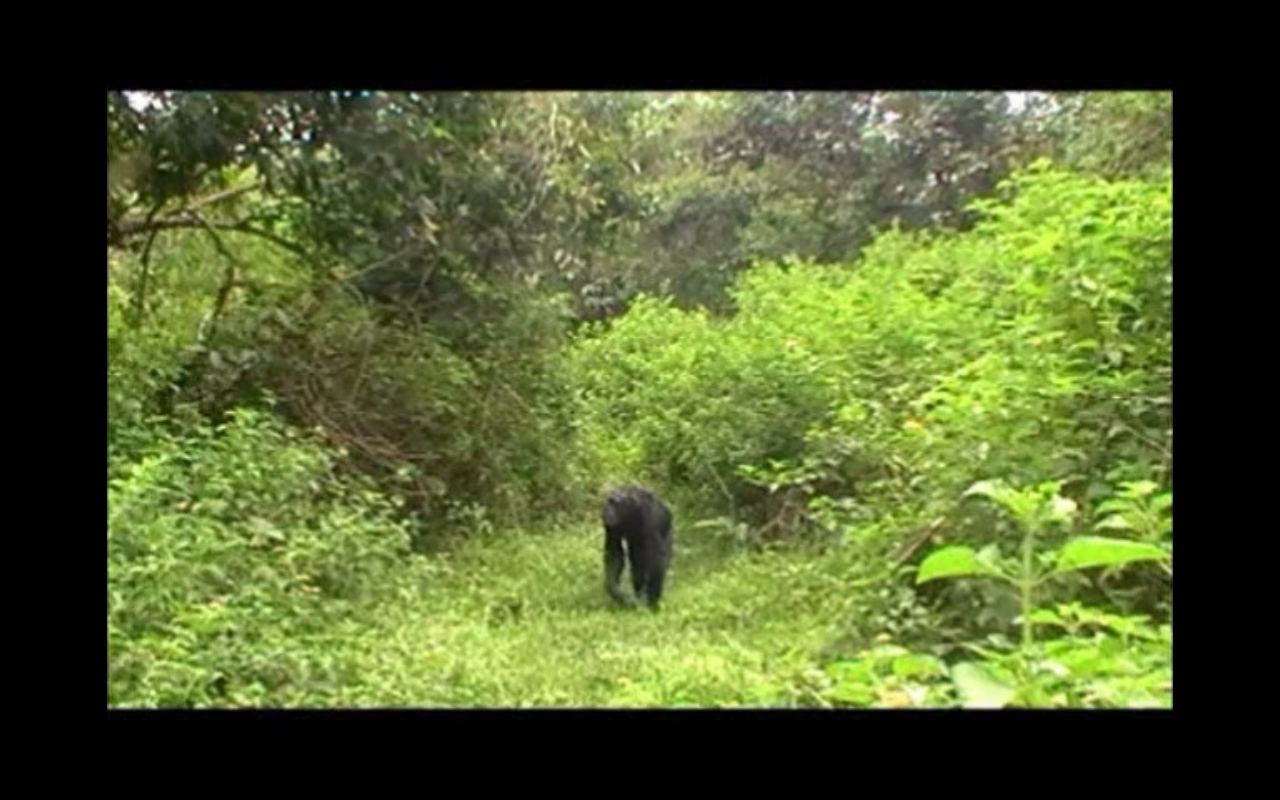
**

**Video S1**. **Video** **illustrating discovery of the moving snake and soft huu production.** Video is filmed from position 1 illustrated in Figure S2 in File S1. Focal adult female, Nambi produces a startle response and then begins producing soft huus (commentated by the observer as ‘huuing’) and visually examining the snake, from a bipedal stance. Nambi’s gaze remains fixated on the snake during the soft huu production.


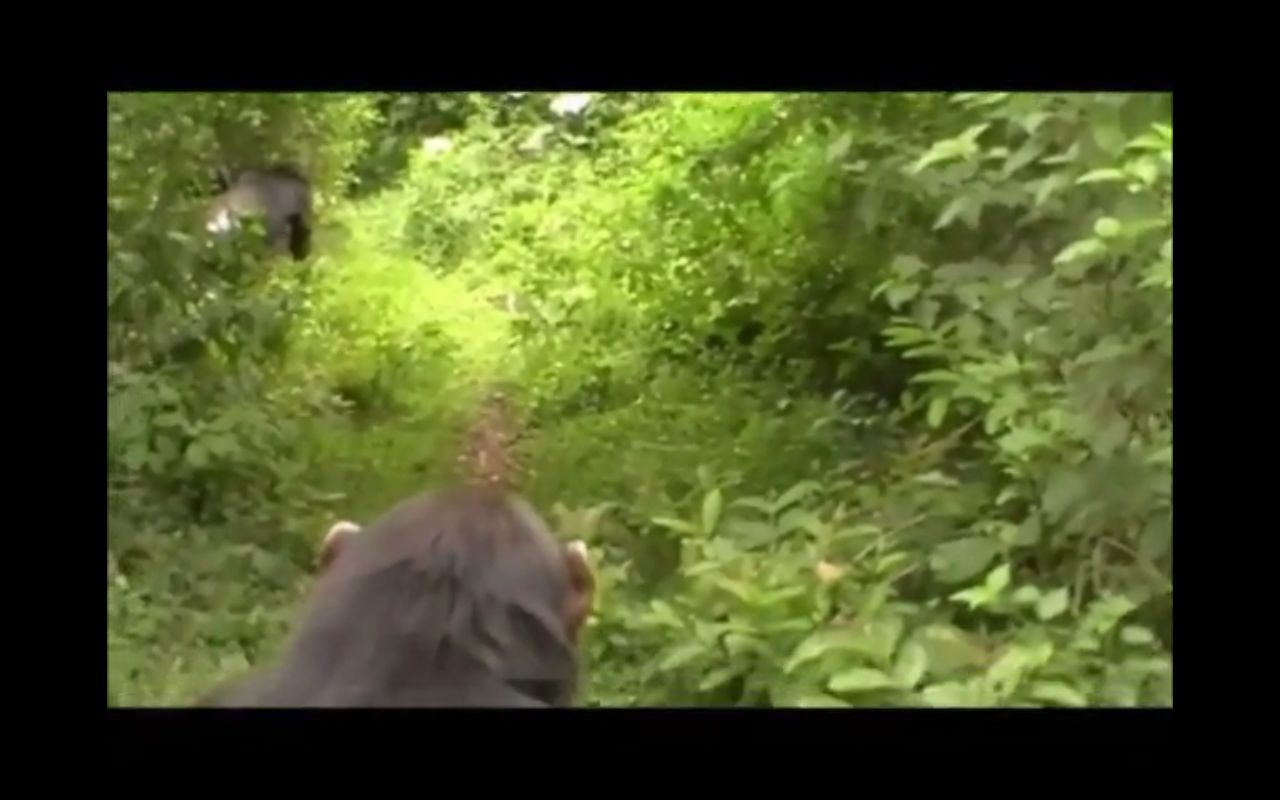


**Video S2**. **Video illustrating gaze alternation and looking at a group member before producing waa barks.** Video is filmed from position 2 illustrated in Figure S2 in File S1. Focal adult female Nambi reacts to the arrival of her adult son Musa by turning and looking at Musa before producing her first waa barks of the trial. Nambi then looks immediately back at the snake, showing gaze alternation between the recipient and the snake whilst calling. During Nambi’s waa bark production, Musa stands bipedally.

**Audio S1. Sound recording of an example Soft Hoo (SH).**

**Audio S2. Sound recording of an example Alarm Hoo (AH).**

**Audio S3. Sound recording of an example Waa Bark (WB).**
